# Comparison of Tracheal Diameter Measurements on Radiograph Versus Computed Tomography at a Tertiary Care Hospital in Pune, Central India

**DOI:** 10.7759/cureus.13755

**Published:** 2021-03-07

**Authors:** Pranav Ajmera, Niharika Prasad

**Affiliations:** 1 Radiology, Dr DY Patil Medical College, Hospital and Research Center, Pune, IND

**Keywords:** trachea, intubation, radiograph, prospective, normal range, multiple detector computed tomography

## Abstract

Background

Variations in tracheal diameter with respect to factors like age and gender are one of the major factors affecting the size of the endotracheal tube (ETT) preferred in a patient. It is important to pre-determine this figure because a tube of a larger size predisposes the patient for tracheal mucosal ischemia, while one of a smaller size may not ensure adequate oxygen saturation in the patient.

Purpose

We undertook this study to assess the accuracy of radiograph versus computed tomography (CT) and comment on whether a CT should be carried out mandatorily in all patients where intubation is needed.

Materials and methods

The study was undertaken at Dr DY Patil Medical College, Hospital, and Research Center, a tertiary care institute in Pune, India. A total of 217 patients in whom both chest radiograph and chest CT were performed were enrolled in the study and measurements were performed at suitable landmarks which correspond to the position of endotracheal tubes.

Results

The males had a mean age of 44.2 years and females of 41.7 years. The mean X-ray transverse diameter was 15.4 ± 3.2 (SD) mm, mean CT axial transverse diameter was 15.3 ± 3.4 (SD) mm, mean CT sagittal diameter was 14.8 ± 3.7 (SD) mm, and the mean CT coronal diameter was 15.2 ± 3.5(SD)mm.

Conclusions

There was a significant difference in mean X-ray transverse diameter (Low kV), CT axial transverse diameter, CT sagittal diameter, and CT coronal diameter between males and females. Mean values were significantly higher in males as compared to females. There was a significant difference in tracheal diameters for different age groups, irrespective of the modality. Bland-Altman analysis revealed no significant difference between chest radiograph and CT for tracheal diameter measurement.

## Introduction

Variations in tracheal diameter with respect to factors like age and gender are one of the major factors affecting the size of the endotracheal tube (ETT) preferred in a patient. It is important to pre-determine this figure because a tube of a larger size predisposes the patient for tracheal mucosal ischemia, while one of a smaller size may not ensure adequate oxygen saturation in the patient [1,2]. Plain computed tomography (CT) is a highly accurate modality to estimate the overall diameter of the trachea (DT) at that particular instant, whether it is the anteroposterior (AP) or transverse diameter, and whether the diameter is measured in inspiratory or expiratory position. But a CT is costly in comparison to a radiograph. The delineation of different tissues on an X-ray varies with the kilovolts peak and is affected by factors like obesity and is liable to scattering, all of which affect the final quality of radiograph obtained, and hence, the measurements [3,4]. Hence, we undertook this study to assess the accuracy of radiograph versus CT in all those cases in which both were performed. An analysis of the accuracy would be useful in determining whether a CT should be carried out mandatorily in all patients where intubation is needed and CT is possible, in order to ensure fewer complications post-intubation.

## Materials and methods

This was a prospective study performed at Dr DY Patil Medical College, Hospital, and Research Center, a tertiary care institute in Pune, India, over a 12-month period between May 2019 to May 2020. As a part of the study, only those patients were enrolled who had undergone both chest radiography and chest CT before being intubated. However, amongst this group, only those patients were analysed who were not suffering from any prior airway diseases, such as chronic obstructive pulmonary disease or having an acute exacerbation of any airway disease, as the inflammation process narrows the inner tracheal diameter. Also excluded were patients who had undergone any thoracic procedure or chest surgery in the previous three months, patients already having an indwelling ETT or airway in-situ, and patients with a sub-optimal radiograph that is either over- or underexposed.

The CT was performed on a 128-slice Philips Ingenuity CT scanner (Philips Medical Systems Nederland B.V., Eindhoven, The Netherlands), while the radiographs were acquired on a 500 mA (milliampere) machine with settings maintained at 55 kV (kilovolts), except in cases where the patient was obese, where it was kept at 60 kV.

Comparisons were made using standard anatomical landmarks, which have been used in previous studies, like the one performed by Sakuraba et al. [2]. For CT, this implied measuring the transverse diameter at the level of the body of the seventh cervical vertebra. In the current study, measurements were also drawn for the coronal and sagittal diameter; these measurements were made after three-dimensional reconstruction of the image at the same level. This was ensured by utilising the triangulating tool to identify the exact point on the axial image in coronal and sagittal sections. On the radiograph images, the transverse diameter was measured by drawing a hypothetical line at the level of bilateral sternal ends of the clavicle. These specific landmarks correspond to the location where the inflated cuff of ETT contacts the mucosa.

Statistical analysis

Data was analyzed using SPSS version 22 software (IBM Corp., Armonk, NY, USA). Categorical data was represented in the form of frequencies and proportions. Continuous data was represented as mean and standard deviation.

The independent t-test was used as a test of significance to identify the mean difference between two quantitative variables. ANOVA (analysis of variance) was the test of significance to identify the mean difference between more than two groups for quantitative data. Pearson correlation was done to find the correlation between two quantitative variables [5-7].

Bland-Altman plot was used to describe the agreement between the two quantitative measurements. P-value (the probability that the result is true) of <0.05 was considered as statistically significant.

## Results

The study included 217 patients, among whom 120 were males and 97 were females The males had a mean age distribution of 44.2 years, and the females had a mean of 41.7 years. A comparison of the mean transverse diameter on radiograph and mean diameter on CT axial view between the two genders revealed a mean figure of 16.3 mm (SD = 3.6 mm) in males and 14.3 mm (SD = 2.2 mm) in females for the radiograph. In the case of CT, the mean figure for axial diameter was 16.7 mm (SD = 3.4 mm) in males and 13.6 mm (SD = 2.5 mm) in females; for sagittal diameter, it was 16.3 mm (SD = 3.7 mm) in males, 13 mm (SD = 2.7 mm) in females; for coronal diameter, it was 16.5 mm (SD = 3.3 mm) in males, 13.5 mm (SD = 2.8 mm) in females.

In the study, among males, there was a significant difference in mean X-ray transverse diameter (low **kV**), CT axial transverse diameter, CT sagittal diameter, and CT coronal diameter with respect to age distribution. Mean X-ray transverse diameter was highest among subjects in the age group 50 to 59 years; mean CT axial transverse diameter was highest among subjects in the age group 70 to 79 years; mean CT sagittal diameter was highest among subjects in the age group 70 to 79 years and 50 to 59 years; and mean CT coronal diameter was highest among subjects in the age group 70 to 79 years (Table 1).

**Table 1:** Mean transverse, coronal, and sagittal tracheal diameters in normal subjects by age for males ^#^ANOVA (analysis of variance)

		X-ray transverse diameter (Low kV)		CT axial transverse diameter		CT saggital diameter		CT coronal diameter	
		Mean	SD	Mean	SD	Mean	SD	Mean	SD
Age	<10 years	8.6	2.0	8.7	2.0	8.6	2.5	9.1	2.3
	10 to 19 years	16.0	1.2	15.0	1.9	15.4	1.9	15.0	2.4
	20 to 29 years	15.9	3.0	15.7	2.4	16.2	3.4	15.8	2.6
	30 to 39 years	16.5	2.2	16.3	2.0	16.9	3.1	16.2	2.6
	40 to 49 years	18.0	2.1	16.8	1.7	16.9	3.0	16.5	1.9
	50 to 59 years	18.2	4.0	18.1	3.2	17.5	3.2	18.0	3.1
	60 to 69 years	16.2	2.2	17.9	1.4	16.7	3.7	17.7	2.4
	70 to 79 years	16.8	3.5	19.0	2.5	17.5	2.8	18.6	2.0
	P-value^#^	<0.001*		<0.001*		<0.001*		<0.001*	

In the study among females, there was a significant difference in mean X-ray transverse diameter (low kV), CT axial transverse diameter, CT sagittal diameter, and CT coronal diameter with respect to age distribution. Mean X-ray transverse diameter was highest among subjects in the age group 70 to 79 years; mean CT axial transverse diameter was highest among subjects in the age group 50 to 59 years; mean CT sagittal diameter was highest among subjects in the age group 40 to 49 years; and mean CT coronal diameter was highest among subjects in the age group 40 to 49 years (Table 2).

**Table 2:** Mean transverse, coronal, and sagittal tracheal diameters in normal subjects by age for females ^#^ANOVA (analysis of variance) kV: kilovolts

		X-ray transverse diameter (low kV)		CT axial transverse diameter		CT sagittal diameter		CT coronal diameter	
		Mean	SD	Mean	SD	Mean	SD	Mean	SD
Age	<10 years	8.7	0.2	7.9	0.9	7.4	2.5	8.3	3.3
	10 to 19 years	12.8	1.7	13.3	1.5	12.5	1.9	12.9	1.3
	20 to 29 years	14.2	1.9	13.4	2.2	13.2	3.1	13.4	2.4
	30 to 39 years	14.8	1.8	13.2	1.6	11.9	2.0	12.7	1.4
	40 to 49 years	13.9	1.9	14.9	2.5	14.0	2.5	15.6	3.5
	50 to 59 years	15.2	1.2	15.3	2.0	13.7	1.5	13.8	3.0
	60 to 69 years	14.9	3.5	13.2	3.3	13.7	2.4	13.7	3.7
	70 to 79 years	15.3	1.3	14.0	2.9	13.5	3.3	14.0	3.2
	P-value^#^	<0.001*		0.007*		0.031*		0.038*	

Overall, the mean X-ray transverse diameter was 15.4 ± 3.2 (SD) mm, mean CT axial transverse diameter was 15.3 ± 3.4 (SD) mm, mean CT sagittal diameter was 14.8 ± 3.7 (SD) mm, and mean CT coronal diameter was 15.2 ± 3.5 (SD) mm.

**Figure 1:**
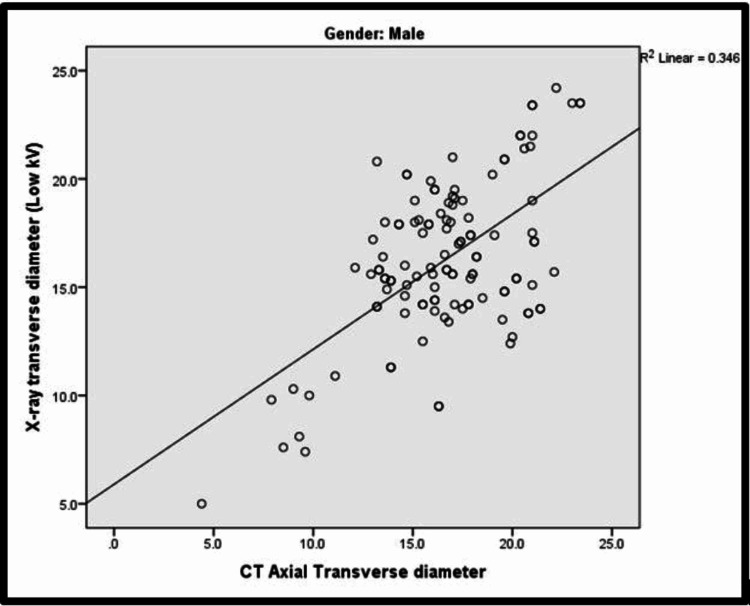
Scatter plot showing correlation between X-ray transverse diameter and CT axial transverse diameter among males kV: kilovolts

**Figure 2:**
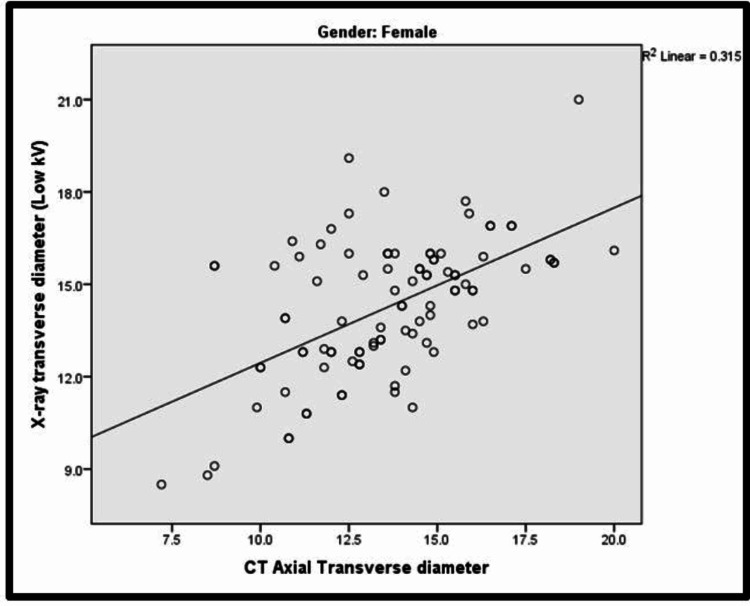
Scatter plot showing correlation between X-ray transverse diameter and CT axial transverse diameter among females kV: kilovolts

In the study, among both males and females, there was a significant positive correlation between X-ray transverse diameter and CT axial transverse diameter, that is, with an increase in X-ray transverse diameter, there was an increase in CT axial transverse diameter and vice versa. The Pearson correlation findings for CT axial transverse diameter in males and females were 0.588 and 0.561, respectively. Corresponding scatter plots for males and females have been depicted in Figure 1 and Figure 2, respectively.

**Figure 3:**
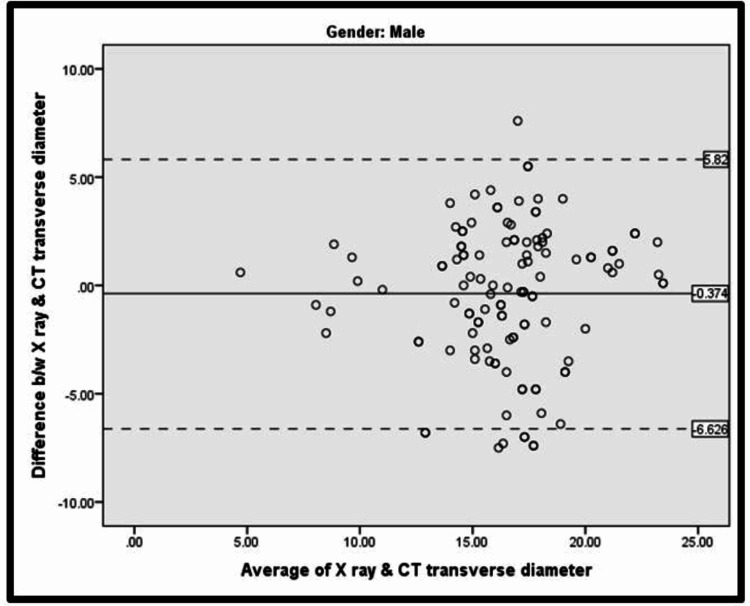
Bland-Altman plot for X-ray vs CT transverse diameter among males The solid line shows bias, and the dotted lines show the limit of agreement.

The Bland-Altman analysis for chest radiograph versus CT transverse diameter among males as depicted in Table 3 and Figure 3 revealed that the mean (difference between X-ray and CT transverse diameter) was -0.374 ± 1.96 (3.191) and the mean (difference between X-ray and CT transverse diameter) ±1.96 SD was -6.626 to 5.878.

**Table 3:** Bland-Altman analysis for X-ray vs CT transverse diameter among males Mean difference between X-ray and CT transverse diameter was -0.374 ± 1.96 (3.191) Mean (difference between X-ray and CT transverse diameter) ±1.96 SD = -6.626 to 5.878

	N	Mean	SD
Difference between X-ray and CT transverse diameter	120	-0.374	3.191
Average of X-ray and CT transverse diameter	120	16.469	3.129

**Figure 4:**
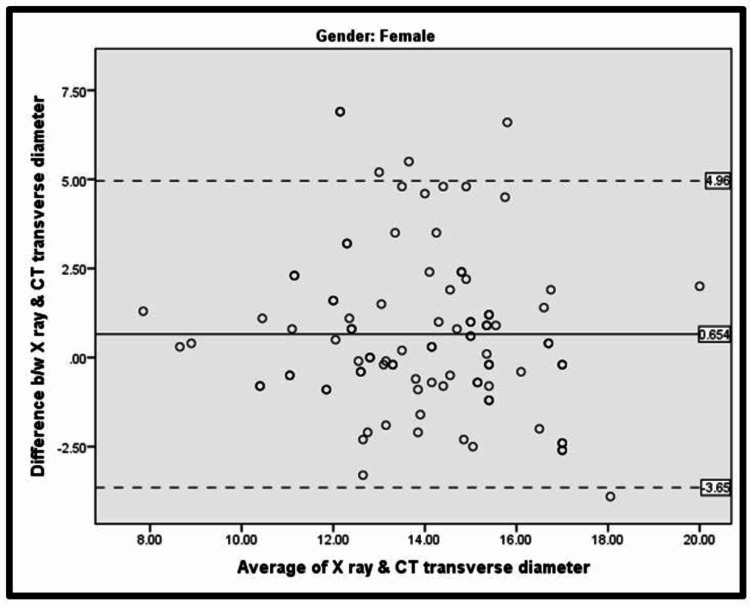
Bland-Altman plot for X-ray vs CT transverse diameter among females The solid line shows bias, and the dotted lines show the limit of agreement.

Among females (Table 4, Figure 4), the Bland-Altman analysis revealed that the mean (difference between X-ray and CT transverse diameter) was 0.654 ± 1.96 (2.231) and the mean (difference between X-ray and CT transverse diameter) ± 1.96 SD was 3.658 to 4.96.

**Table 4:** Bland-Altman analysis for X-ray vs CT transverse diameter among females Mean (difference between X-ray and CT transverse diameter) was 0.654 ± 1.96 (2.231) Mean (difference between X-ray and CT transverse diameter) ±1.96 SD = 3.658 to 4.96

	N	Mean	SD
Difference b/w X ray & CT transverse diameter	97	0.654	2.231
Average of X ray & CT transverse diameter	97	13.942	2.095

## Discussion

The knowledge of the transverse diameter of the trachea at the level where the cuff is inflated, allows the determination of the size of the endotracheal tube to be inserted and, in turn, avoid discomfort to the patient in the form of pain and hoarseness. It also avoids complications like pressure necrosis and tracheal rupture [8-11]

While data exists on the normal values for coronal and sagittal diameters, these figures are relatively old and were mostly determined utilising radiographs only. With the advent of CT, more accurate measurements can be acquired. In fact, CT happens to be the most powerful technique and gives the most superior resolution [12] in-vivo compared to other available options. Other non-invasive modalities that can be utilised to take measurements of the trachea include endotracheal-ultrasonography (EUS), magnetic resonance imaging (MRI), and bronchoscopy. CT has an advantage over the rest as EUS is operator- and technique-dependent to some extent, MRI is too costly to be practically feasible in most cases, and bronchoscopy is an invasive procedure.

In our study, we found that there was a significant difference in mean X-ray transverse diameter (low kV), CT axial transverse diameter, CT sagittal diameter, and CT coronal diameter between males and females. Mean values were significantly higher in males as compared to females. This is similar to the results published by Mihara et al. [13] and Breatnach et al. [14] who found that both the upper and lower limit of normal in males is more than in females.

We also found that there is a significant difference in tracheal diameters for different age groups, irrespective of the modality the measurements were performed on. This is in concordance with the study performed by Mihara et al. [13]. The study though was limited in the aspect that this was a single-centre study. In future, more such studies from different parts of India should be carried out to account for differences between various regions due to environment and genetics.

## Conclusions

Bland-Altman analysis revealed that the transverse diameters, whether measured on a radiograph or on a CT, were comparable to each other as most of the values were falling within the limit of agreement, except a few outliers. Hence, this implies that a radiograph, being the cheaper alternative to CT, should be preferred as there is no significant difference in tracheal dimensions measurement. The limitations of our study were that the entire patient data was collected from a single, tertiary institute, and the observations were drawn by a single radiologist with five years of experience.
